# Retraction: Androgen receptor CAG and GGN repeat length variation contributes more to the tumorigenesis of osteosarcoma

**DOI:** 10.18632/oncotarget.17877

**Published:** 2017-05-15

**Authors:** Yongxiang Shi, Weishan Chen, Qinghuai Li, Zhaoming Ye

**Present:** Due to the authors’ negligence during the preparation of this manuscript for submission, it contains a plethora of data errors. Authors sincerely apologize for their mistakes and wish to retract their manuscript.

**Correct:** The online version of the original article can be found under doi.

Original article: Oncotarget. 2016; 7:68151-68155. doi: 10.18632/oncotarget.11902

